# Supplementary material for: “We Work in an Industry Where We’re Here to Care for Others, and Often Forget to Take Care of Ourselves”: Aged-Care Staff Views on Self-Care
Source: Geriatrics (Basel). 2025 Jan 2;10(1):3. doi: 10.3390/geriatrics10010003 (PMC11755575; doi:10.3390/geriatrics10010003)
Supplement: Supplementary file 1 [file geriatrics-10-00003-s001.zip › geriatrics-3239396-supplementary/geriatrics-3239396-supplementary.pdf]

Supplementary Material: S1: Interview Guide

Topic: What is 'self-care' and why does it matter to aged care staff?

Self-care is difficult to define. There are several formal definitions such as these produced by the World Health Organisation and academics. But these definitions were crafted from evidence collected in different settings, such as primary health care, or with different population groups, such as doctors or surgeons, and they may not be meaningful to you as an aged care worker. We want to explore what you think about this idea or self-care. If we have a better understanding about what self-care means to people working in aged care, what problems you face, or needs that you have, then we can tailor a resource to your specific context and needs.

- As an aged care worker, what do you think about self-care?
- Do you think self-care matters for aged care workers? Why?

Topic: What resources have been developed for aged care staff, and can staff benefit from them?

Many people believe that self-care matters for aged care workers. They argue that a career in aged care is a rewarding experience, but it can also sometimes be emotional and stressful, and if workers don't take care of themselves, they will burnout and their ability to care for others will be impaired. So, some organisations or peak bodies have developed resources to help aged care staff in taking care of themselves. Now, I'm going to show you some of these resources and I'd like to know what you think about them.

- Do you think you or your colleagues can benefit from resources like these? Why?
- What do you like about them?
- What don't you like about them?
- What would you do to improve them?

Topic: What are some ideas for managing stress and maintaining wellbeing over the long term while doing this work?

We want to develop an online self-care resource for aged care staff. We know that aged care work is challenging. You face a whole raft of demands on your job, you have the ongoing threat of COVID-19. And we think that death and dying of residents/clients is also one of those stressors. So, we want to learn more about how you manage that stress, and also how you maintain a good level of wellbeing over the long term, or even thrive doing this work. Then perhaps we can take what we learn from you and translate that into an online resource.

Can you think about the past month at work:

- what kind of things made you feel good? If might be something you did or something that someone else did.
- what things made you feel not so good or terrible?

Now, can you think about the last time a resident/client you cared for died:

- how did you feel?
- what kind of things helped you to deal with this experience?
- what things made didn’t help?

Now, can you imagine that you have a new colleague at work, and you are responsible for their wellbeing:

- what would you do?
- what do you think they would need?
